# Association of VAV2 and VAV3 polymorphisms with cardiovascular risk factors

**DOI:** 10.1038/srep41875

**Published:** 2017-02-03

**Authors:** Nuria Perretta-Tejedor, Javier Fernández-Mateos, Luis García-Ortiz, Manuel A. Gómez-Marcos, José I. Recio-Rodríguez, Cristina Agudo-Conde, Emiliano Rodriguez-Sánchez, Ana I. Morales, Francisco J. López-Hernández, José M. López-Novoa, Rogelio González-Sarmiento, Carlos Martínez-Salgado

**Affiliations:** 1 Translational Research on Renal and Cardiovascular Diseases (TRECARD), Department of Physiology and Pharmacology, University of Salamanca, Salamanca, Spain; 2 Institute of Health Sciences Studies of Castilla y Leon (IECSCYL), Research Unit, University Hospital of Salamanca, Spain; 3 Institute of Biomedical Research of Salamanca (IBSAL), Salamanca, Spain; 4 Molecular Medicine Unit, Department of Medicine, University of Salamanca and Institute of Molecular and Cellular Biology of Cancer (IBMCC), University of Salamanca-CSIC, Salamanca, Spain; 5 Research Unit, Primary Care Center of La Alamedilla, Salamanca, Spain; 6 Renal and Cardiovascular Pathophysiology Unit, Institute Reina Sofia of Nephrological Research, Department of Physiology and Pharmacology, University of Salamanca, Salamanca, Spain

## Abstract

Hypertension, diabetes and obesity are cardiovascular risk factors closely associated to the development of renal and cardiovascular target organ damage. VAV2 and VAV3, members of the VAV family proto-oncogenes, are guanosine nucleotide exchange factors for the Rho and Rac GTPase family, which is related with cardiovascular homeostasis. We have analyzed the relationship between the presence of VAV2 rs602990 and VAV3 rs7528153 polymorphisms with cardiovascular risk factors and target organ damage (heart, vessels and kidney) in 411 subjects. Our results show that being carrier of the T allele in VAV2 rs602990 polymorphism is associated with an increased risk of obesity, reduced levels of ankle-brachial index and diastolic blood pressure and reduced retinal artery caliber. In addition, being carrier of T allele is associated with increased risk of target organ damage in males. On the other hand, being carrier of the T allele in VAV3 rs7528153 polymorphism is associated with a decreased susceptibility of developing a pathologic state composed by the presence of hypertension, diabetes, obesity or cardiovascular damage, and with an increased risk of developing altered basal glycaemia. This is the first report showing an association between VAV2 and VAV3 polymorphisms with cardiovascular risk factors and target organ damage.

Hypertension (HT), diabetes mellitus (DM) and obesity are the most common risk factors associated to the development of cardiovascular diseases^1^,^2^, the main cause of death worldwide^3^. HT is directly related to the incidence of cardiovascular complications (heart attack, ictus or peripheral arterial disease). Moreover, high blood pressure (BP) or DM together with other risk factor maximize their cardiovascular risk^1^. The presence of renal^4^, cardiac^5^ and vascular^6^,^7^ target organ damage (TOD) also increases cardiovascular risk^8^. Vascular damage affects large and small vessels: Damage in large vessels is associated with atherosclerosis whereas damage in small vessels (narrowing or blocked retinal blood vessels, macular edema) appears in disorders such as retinopathy, as reported in some studies which associate retinal vessels caliber with arterial hypertension^9^, left ventricular hypertrophy (LVH)^10^, metabolic syndrome^11^, cerebrovascular accident^12^ and coronary diseases^13^ and cardiovascular risk.

The guanine nucleotide exchange factors VAV2 and VAV3 are members of the VAV family of proto-oncogenes. Both are GDP-GTP dependent factors that stimulate Rho and Rac GTPases^14^, which have a similar structure, leading to the activation of intracellular pathways involved in cytoskeletal organization, transcriptomal dynamics and other biological responses^15^. VAV proteins are expressed in a variety of tissues^14^,^16^ and in hematopoietic cells^17^ and participate in the development and function of hematopoietic lineage cells, lymphocytes, neutrophils, natural killers and osteoclasts^15^,^18^,^19^. All VAV family members contains an N-terminal calponin homology (CH) domain, a catalytic Dbl-homology (DH) domain, an acidic (Ac) region, a pleckstrin-homology (PH) region, a C1 subtype zinc finger (ZF) domain, a SH2 and a C-terminal SH3 (CSH3), a proline-rich region (PRR) and a more N-terminally-located SH3 (NSH3)^14^.

Sauzeau *et al*.^20^ analysed the effects of the absence of VAV2 in knock-out mice, which showed cardiovascular alterations such as cardiovascular remodeling, thickening of aorta media walls and LVH with fibrosis. In addition, these mice showed renal fibrosis, tachycardia and HT due to hyperstimulation of the renin/angiotensin/aldosterone axis and sympathetic nervous system^20^. They also observed a similar phenotype in VAV3^−/−^ mice (cardiovascular remodeling, HT, tachycardia, kidney and heart fibrosis)^21^, thus suggesting that both VAV2 and VAV3 play a key role in cardiovascular homeostasis^20^,^21^. On the other hand, Menacho-Marquez *et al*.^22^ have related VAV3 with the metabolic syndrome and obesity, as VAV3^−/−^ mice fed with chow diet developed metabolic syndrome, whereas these mice fed under fatty diets showed resistance against obesity and metabolic syndrome^22^.

Although several studies have observed that some single nucleotide polymorphisms (SNPs) in VAV genes are related to pathologies such as schizophrenia (VAV3 rs1410403)^23^, hypothyroidism (VAV3 rs4915077)^24^ and candidate genes for spontaneous glaucoma (VAV2 rs2156323 and VAV3 rs2801219)^25^- albeit later studies seem to rule out this involvement^26^, there are not studies relating VAV polymorphisms with cardiovascular risk. Thus, our aim is to analyze the relationship between VAV2 (rs602990) and VAV3 (rs7528153) polymorphisms with cardiovascular risk factors and TOD (heart, vessels and kidney) in a Spanish population^8^.

## Results

We analyzed 411 subjects. Tables 1 and 2 summarize clinical, demographic and physical variables of the subjects under the study. The distribution of genotypes of VAV2 rs602990 and VAV3 rs7528153 polymorphisms in control samples are in Hardy-Weinberg equilibrium (Table 3).

There are statistically significant differences in the genotypic distribution for VAV2 rs602990 or VAV3 rs7538153 polymorphisms in cardiovascular risk parameters such as the presence of cardiovascular risk factors, TOD, altered basal glycaemia or obesity.

On the one hand, there are statistically significant differences in the VAV3 rs7538153 genotypic distribution between the patients group (n = 281) and controls (n = 130). Being carrier of the T allele is associated with a decreased susceptibility of developing hypertension, diabetes, obesity or cardiovascular damage in the dominant model (Table 4). There are also significant differences in the genotypic distribution for VAV3 rs7538153 SNP between patients with (n = 51) and without altered basal glycaemia (n = 360). Being carrier of the T allele confers an increased risk of developing altered basal glycaemia in the recessive model (Table 5).

On the other hand, there are statistically significant differences in the allelic distribution for VAV2 rs602990 polymorphism between males with TOD (n = 107) and controls (n = 93). Being carrier of the T allele is associated with an increased risk of TOD in the dominant model (Table 6). There are also differences in VAV2 rs602990 SNP genotypic distribution between obese (BMI > 30) (n = 123) and non-obese (BMI 25–30) patients (n = 197) in the recessive model, where being carrier of the T allele is associated with an increased risk of obesity (Table 7) and a decreased average and minor retinal artery caliber (Table 8). Being carrier of the T allele in this SNP is also associated with low levels of DBP and low levels of right ABI (Table 8).

## Discussion

Some polymorphic variants of genes involved in cardiovascular regulation seem to influence the risk or predisposition to developing cardiovascular disease. For example, a nitric oxide synthase 3 (NOS3) polymorphism is related to rapid progression of heart failure in patients with dilated cardiomyopathy^27^. Moreover, polymorphic variants of the endothelial nitric oxide synthase (eNOS) gene are associated with coronary artery disease^28^. On the other hand, a paraoxonase 1 polymorphism is related with low-density lipoprotein cholesterol levels and with the risk of premature myocardial infarction, whereas another polymorphism of the same gene is associated with a positive family history of coronary artery disease^29^. Polymorphisms in angiotensinogen, angiotensin converting enzyme, angiotensin II type I receptor and aldosterone synthase, as well as polymorphisms in paraoxonase 2, NOS3 endothelin-1 and α and β-adrenergic receptors, are related to essential hypertension, myocardial stiffness and infarction, heart failure, coronary artery atherosclerosis, idiopathic dilated cardiomyopathy and left ventricle hypertrophy^30^.

Our study shows an association between VAV2 rs602990 and VAV3 rs7528153 polymorphisms with several cardiovascular risk factors and TOD (heart, vessels and kidney) in a Spanish population. Hypertension, diabetes and obesity are the most common cardiovascular risk factors^1^,^2^, closely associated to the development of cardiovascular diseases, inducing renal and cardiovascular TOD^1^,^8^. VAV genes have been related with cardiovascular homeostasis^20^,^21^. Moreover, SNPs in VAV genes have been related with several diseases such as schizophrenia, hypothyroidism and glaucoma^23^,^24^,^25^. The SNP T298A VAV3 rs7528153 is located in the DH domain, which catalyzes the activation of Rho and Rac GTPases^31^. There is an association between this VAV3 rs7528153 polymorphism and the presence of a pathologic state by the presence of hypertension, diabetes, obesity or cardiovascular disease (or combinations thereof), as T allele carriers of this SNP have a decreased risk of developing this pathologic state. VAV3^−/−^ mice show cardiovascular alterations such as hypertension, tachycardia or cardiovascular remodeling^21^ and metabolic syndrome^22^ due to a chronic sympathetic excitation. In the ventrolateral medulla, a brainstem area that modulates respiratory, sympathetic and cardiovascular activities^32^, VAV3 acts regulating the conduction of axons of GABAergic neurons from the caudal to the rostral region^33^. The lack of VAV3 confers a reduced GABAergic transmission between these two areas leading to chronic sympathetic excitation^33^ and a variety of sympathetic mediated defects (cardiovascular defects, hypertension or tachycardia). Therefore, it is known that VAV3 has an important role contributing to proper axon guidance^33^ that are under the control of Rho/Rac family proteins^34^. Thus, we suggest that the SNP in the DH domain (rs7538153) confers an increased catalytic activity of Rho/Rac proteins in the GABAergic neurons, which contributes to a proper axon guidance and therefore a correct sympathetic activation.

We have found an association between VAV3 rs7528153 polymorphisms and altered basal glycaemia. Being carrier of the T allele is associated with increased risk of higher values of basal glycaemia. AMP-activated protein kinase (AMPK) acts as an upstream signal for VAV3 induction involving metformin-mediated signaling. Loss of VAV3 potentiates metformin-mediated glucose uptake, suggesting that VAV3 could become a molecular target for blood glucose regulation through AMPK^35^. We suggest that this SNP may affect the metformin-mediated glucose uptake through AMPK. In addition, as VAV3 is down-regulated in human pancreas with type 2 diabetes compared to healthy pancreas, this polymorphic variant of VAV3 may contribute to less severe diabetic neurophaties^36^.

The SNP V594M VAV2 rs602990 is located in the first SH3 domain. We detected an association between VAV2 rs602990 polymorphisms and the presence of TOD. Being carrier of the T allele confers an increased risk of TOD in males (increased PP, HVI, IMT, PWV, ABI or microalbuminuria). Sauzeau *et al*. observed that VAV2^−/−^ mice showed cardiovascular and renal alterations, such as remodeling in the cardiovascular system, LVH with fibrosis, interstitial renal fibrosis and lower glomerular filtration rate^21^ due to a chronic sympathetic excitation. In addition, double VAV2^−/−^, VAV3^−/−^ and triple VAV1^−/−^, VAV2^−/−^ and VAV3^−/−^ mice showed defects in dendritic spine development, in axon transmission and synaptic plasticity in cortical, renal and hippocampal neurons due to deregulation of Rac activity^37^,^38^. We suggest that this SNP in the SH3 domain (which have a great affinity for proline rich sequences and recognize proteins in specific cell regions^14^) reduces the catalytic activity of VAV2 (regulating Rac) in cortical, retinal an hippocampal neurons. Therefore, it produces a defect in axon guidance, which implicates a chronic sympathetic excitation as well as cardiovascular and renal alterations.

ABI index is an indicative parameter of peripheral arterial disease and a low ABI index is directly related with vascular damage^1^. According with our data, T allele in VAV2 rs602990 polymorphism is associated with low levels of right ABI, and therefore is associated with an increased risk of vascular damage. Moreover, T allele in VAV2 rs602990 polymorphisms is also associated with an increased risk of obesity. On the other hand, the T allele in this VAV2 SNP is associated with reduced retinal artery caliber. Retinal arterial narrowing is associated with retinal damage such as retinopathy. Several studies found a relationship between retinal vessels caliber with LVH^10^, metabolic syndrome^11^, cerebrovascular accidents^12^ and coronary diseases^13^.

Although Sauzeau *et al*. observed that VAV2^−/−^ mice were hypertensive due to sympathetic excitation, some studies reported that Rac1 is involved in vasoconstriction^39^. Overexpression of Rac1 in smooth muscle cells in mice induces hypertension as it regulates the redox state of the blood vessels and blood pressure homeostasis through the NADPH oxidase pathway^40^. VAV2 is a guanosine nucleotide exchange factor which activates Rac1 in many cell types^41^,^18^. In our study, we found that being carrier of the T allele in VAV2 rs602990 polymorphism is associated with lower levels of DBP, suggesting that this polymorphism confers less catalytic activity to VAV2, resulting in less activation of Rac 1 and therefore low DBP.

Overall, this study shows the association between some VAV2 and VAV3 polymorphisms with cardiovascular risk factors in a Spanish population. However, this study has some limitations. First, this is a retrospective study. Second, due to the number of analyzed patients, there is a limitation in the statistical power of the study. Thus, in order to confirm our results, studies should be done in a larger population.

In conclusion, this is the first study showing the association between VAV2 and VAV3 polymorphisms with cardiovascular risk factors and TOD, possibly through their influence in Rho and Rac pathways. These polymorphisms are associated with indicators frequently used in the clinical practice to evaluate cardiovascular damage (ABI, retinal artery thickness, DBP, basal glucose and integrated cardiovascular pathologic state). Thus, our study suggests that the analysis of VAV2 and VAV3 polymorphisms might be a useful diagnostic and prognostic marker of susceptibility to cardiovascular damage.

## Methods

This is a cross-sectional study performed in patients with or without DM and HT. 411 subjects aged between 20–80 years were recruited in the Primary Care Research Unit of La Alamedilla Health Centre, Salamanca (Spain), covering a population of 46,000 inhabitants. HT was diagnosed when the mean of three different BP measurements was ≥120 mm Hg for systolic blood pressure (SBP) or ≥80 mm Hg for diastolic blood pressure (DBP) or when patients received antihypertensive drugs. DM was diagnosed following 2 criteria: basal plasma glucose ≥126 mg/dL, glycosylated hemoglobin (HbA1c) > 6,5% or when patients received antidiabetic drugs. Obesity was diagnosed by body mass index (BMI) ≥ 30 kg/m^2^ 1. Dyslipidemia was considered when total cholesterol >4.9 mmol/L (190 mg/dL) or low density lipoprotein cholesterol >3 mmol/L (115 mg/dL) or high-density lipoprotein cholesterol: men <1.0 mmol/L (40 mg/dL), women <1.2 mmol/L (46 mg/dL) or triglycerides >1.7 mmol/L (150 mg/dL). In this study we consider pathologic state when there is a diagnostic of HT, DM, obesity or cardiovascular disease (ischaemic stroke or cerebrovascular disease). Exclusion criteria: patients unable to comply with the protocol requirement (psychological and/or cognitive disorders, failure to cooperate, educational limitations and problems in understanding written language, and failure to sign the informed consent document), patients participating in a clinical trial or patients with serious comorbidities. Normotensive and normoglycemic patients with the above described exclusion criteria and without detectable renal and cardiovascular alterations were selected as controls.

TOD is a variable composed of renal TOD (assessed by microalbuminuria) vascular TOD (assessed by carotid intima-media thickness (IMT), pulse wave velocity (PWV) and ankle-brachial index (ABI)) and cardiac TOD (assessed by left ventricular hypertrophy (LVH) and pulse pressure (PP)).

### Ethical and legal issues

The experimental protocol was in accordance with the Declaration of Helsinki (2008) of the World Medical Association, approved by the University Hospital of Salamanca Ethics Committee and complied with Spanish data protection law (LO 15/1999) and specifications (RD 1720/2007). An informed consent was obtained from all participants recruited in the study.

### Anthropometric measurements

We calculated BMI (kg/m^2^) measuring body weight using a homologated electronic scale (Seca 70, Hamburg, Germany; precision ± 0.1 kg), and height with a portable system (Seca 222).

### Plasma and urine determinations

We collected urine and blood samples in the morning after fasting for at least 8 hours. We measured creatinine, basal glucose, HbA1c, triglycerides, high-density lipoprotein (HDL)-cholesterol, low-density lipoprotein (LDL)-cholesterol in plasma, and creatinine and microalbumin in urine using standard automatic techniques on a blind basis in the Biochemistry laboratory of the University Hospital, Salamanca (Spain).

### Blood pressure determination

Office BP evaluation involved three measurements of SBP and DBP with a validated OMRON model M7 sphygmomanometer (Omron Health Care, Kyoto, Japan) following the recommendations of the European Society of Hypertension (ESH)^1^. We calculated SBP, DBP, and PP with the mean values of the second and third measurements.

### Evaluation of peripheral artery disease

We used the ankle–brachial index (ABI), in patients refrained from drinking coffee or smoking tobacco for at least 8 h before measurements and at 22–24 °C. With the patient lying in supine position and with the feet uncovered, we measured BP in the lower limbs after resting for 20 min using a portable Doppler system Minidop Es-100Vx (Hadeco Inc, Miyamae-ku Kawasaki, Japan). We calculated ABI automatically for each foot by dividing the higher of the two SBP in the ankle by the higher of the two SBP in the arm. An ankle–brachial index <0.9 is considered abnormal^1^.

### Determination of left ventricular hypertrophy

Electrocardiography (ECG) was performed with a General Electric MAC 3.500 ECG System (Niskayuna, New York, USA) that automatically measures wave voltage and duration and estimates the criteria of the Cornell voltage duration product (VDP)^42^ LVH was defined as a Sokolow-Lyon index >3.5 mV; RaVL >1.1 mV, Cornell VDP >244 mV*ms or RaVL >1.1 mV^1^.

### Determination of pulse wave velocity

We evaluated PWV with the SphygmoCor System (AtCor Medical Pty Ltd, Head Office, West Ryde, Australia). We analyzed the pulse waves of the carotid and femoral arteries with the patient in the supine position measuring the delay with respect to the ECG wave, and then calculating PWV. We obtained distance measurements with a measuring tape from the sternal notch to the carotid and femoral arteries at the sensor location.

### Assessment of carotid intima-media thickness

We used a Micromax ultrasound device (SonoSite Inc, Bothell, WA) paired with a 5–10 MHz multifrequency high-resolution linear transducer with Sonocal software for performing automated measurements of carotid IMT in order to optimise reproducibility. We made measurements of the common carotid after examination of a 10 mm longitudinal section at a distance of 1 cm from the bifurcation. We took measurements in the proximal wall, and in the distal wall in the lateral, anterior and posterior projections, following an axis perpendicular to the artery to discriminate two lines: one for the intima-blood interface and the other for the media-adventitious interface. We took six measurements of both the right and left carotid arteries, using average values (average carotid IMT) and maximum values (maximum carotid IMT) automatically calculated by the software^43^. Average IMT was considered abnormal if >0.90 mm or in the presence of atherosclerotic plaques with a diameter of 1.5 mm or a focal increase of 0.5 mm or 50% of the adjacent IMT^1^.

### Evaluation of renal function

We assessed kidney damage measuring the estimated glomerular filtration rate using the Chronic Kidney Disease Epidemiology Collaboration (CKD-EPI)^44^ equation, the Modification of Diet in Renal Disease- Isotopic Dilution Mass Spectrometry (MDRD-IDMS)^45^ and proteinuria, as assessed by the albumin/creatinine ratio following the criteria of the 2013 European Society of Hypertension/European Society of Cardiology Guidelines^1^. We considered subclinical renal damage when glomerular filtration rate was below 30–60 mL/min/1.73 m^2^ or microalbuminuria between 30–300 mg/24 h or albumin–creatinine ratio between 30–300 mg/g, 3.4–34 mg/mmol. We defined renal disease as a glomerular filtration rate <30 mL/min/1.73 m^2^, proteinuria >300 mg/24 h or albumin/creatinine ratio >300 mg/24 h^1^.

### Evaluation of retinopathy

We evaluated retinography with a Topcon TRC NW 200 non-mydriatic retinal camera (Topcon Europe B.C., Capelle a/d Ijssel, The Netherlands), obtaining nasal and temporal images centered on the disk. Images were loaded into our own developed software, AV Index calculator (Ciclorisk SL, Salamanca, Spain, registry no. 00/2011/589), which automatically estimates the mean caliber of veins and arteries in millimetres as an arteriole– venule ratio, arteriovenous index (AVIx). An AVIx of 1.0 suggests that arteriolar diameters are on average the same as venular diameters in that eye, whereas a smaller AVR suggests narrower arterioles. The pairs of main vessels in the upper and lower temporal quadrants were used, rejecting all other vessels, to improve reliability and increase efficiency of the process, analysing measures for each quadrant separately and together to estimate the mean measure in each eye.

### Cardiovascular risk assessment

We estimated cardiovascular risk (CVR) of morbidity and mortality using the 2013 guidelines of the ESH^1^, which stratify cardiovascular risk based on blood pressure, cardiovascular risk factors, asymptomatic organ damage and presence of diabetes, symptomatic cardiovascular disease or chronic kidney disease. The classification in low, moderate, high and very high risk is retained from previews guidelines and refers to the 10-year risk of cardiovascular mortality as defined by the 2012 ESC prevention guidelines 46, based in the SCORE scale, that classifies the cardiovascular risk in low (<1%), moderate (1–5%), higher (5–10%), and very high risk (≥10%).

### DNA isolation and genotyping

We extracted genomic DNA from peripheral blood leukocytes by the phenol-chloroform method^47^. We identified VAV2 rs602990 and VAV3 rs7528153 polymorphisms by the allelic discrimination assay with TaqMan® probes (Life Technologies, Carlsbad, California, USA) (Table 3) using specific oligonucleotides to amplify the regions containing the polymorphisms and two labelled probes with the fluorochromes VIC and FAM to detect both alleles of each polymorphism^48^. We performed the reaction with the Universal PCR Master Mix in the Real-Time PCR system of Step-One Plus (Applied Biosystems, Forster, California, USA). To ensure the reproducibility, a 5% of random samples were re-genotyped.

### Statistical analysis

We used the statistical software SPSS v.21.0 (Armonk, New York, USA). We tested control group subjects for conformity to the Hardy-Weinberg equilibrium using the chi-squared test for each polymorphism. We analysed the association between the different clinical and molecular qualitative variables by cross tabs and the Pearson *X*^*2*^test. The odds ratio (OR) and 95% confidence intervals were calculated by a logistic regression model to evaluate the association with the risk to develop the disease. We compared quantitative variables and the influence of polymorphism distribution by the ANOVA test in those cases that followed a parametric distribution (Levene’s test for homogeneity of variances, p > 0.05). When data followed a non-parametric distribution, we applied a Mann Whitney U test. In order to consider confounding variables, we made a statistical adjustment by sex and the continuous variable of age. We considered statistically significant differences when P-value was <0.05.

## Additional Information

**How to cite this article:** Perretta-Tejedor, N. *et al*. Association of VAV2 and VAV3 polymorphisms with cardiovascular risk factors. *Sci. Rep.* **7**, 41875; doi: 10.1038/srep41875 (2017).

**Publisher's note:** Springer Nature remains neutral with regard to jurisdictional claims in published maps and institutional affiliations.

## Figures and Tables

**Table 1:** Characteristics of the 411 patients included in the study.

	N	%
Sex		
Female	192	47.2
Male	215	52.1
HT		
Yes	209	50.9
No	202	49.1
Diabetes Mellitus		
Yes	66	83.5
No	345	16.0
Pathologic state		
Yes	281	31.5
No	130	68.0
Target organ damage		
Yes	191	46.2
No	220	53.3
Altered basal glycaemia		
Yes	51	12.3
No	360	87.2
BMI		
BMI < 25	74	17.9
BMI 25–30	210	50.8
BMI > 30	127	30.8
Dyslipidemia		
Yes	386	93.5
No	24	5.8
Elevated PP		
Yes	97	23.5
No	314	76.0
LVH		
Yes	34	8.2
No	367	88.9
C-IMT		
Yes	73	17.7
No	336	81.4
Altered PWV		
Yes	63	15.3
No	343	83.1
PAD		
Yes	7	1.7
No	402	98.3
CV risk		
<1%	58	14.1
1–5%	198	48.2
5–10%	84	20.4
>10%	71	17.3
Antihypertensive drugs		
Yes	227	55.0
No	184	44.6
Antidiabetic drugs		
Yes	66	16.0
No	345	83.5
Lipid-lowering drugs		
Yes	164	39.7
No	247	59.8

BMI: body mass index; C-IMT: carotid intima media thickness; CV: cardiovascular; HT: hypertension; LVH: left ventricular hypertrophy; PAD: peripheral arterial disease; PP: pulse pressure; PWV: pulse wave velocity.

**Table 2:** Demographic, physical and basic analytical values of the patients included in the study.

Characteristics	N	Average	SD
Weight. Kg	411	76.49	14.57
Age. years	411	60.31	9.79
BMI. Kg/m^2^	411	28.54	4.38
SBP. mmHg	411	133.84	17.18
DBP. mmHg	411	81.31	10.54
PP. mmHg	411	52.53	13.03
Heart rate. beats/min	411	70.11	10.97
ABI left	411	1.14	0.10
ABI right	410	1.13	0.10
Average C-IMT. mm	409	0.74	0.10
Maximun C-IMT. mm	409	0.90	0.12
PWV. m/s	406	8.64	1.61
VDP-Cornell. mV/ms	401	1548.23	565.01
Basal glycemia. mg/dL	411	95.60	28.10
Plasma creatinine. mg/dL	411	0.85	0.19
HDL-cholesterol. mg/dL	398	53.64	14.20
LDL- colesterol. mg/dL	403	132.09	34.32
Triglycerides. mg/dL	411	128.63	73.79
HbA1c. %	402	5.92	0.92
Hemoglobin. g/dL	407	15.09	1.17
Urinary creatinine. mg/dL	404	103.95	52.48
Microalbuminuria. mg/dL	403	13.14	65.61
Left artery. μm	229	108.08	13.31
Right artery. μm	234	109.04	12.57
Average artery. μm	261	109.16	12.13
Minor artery. μm	261	104.83	13.09
Left vein. μm	229	141.42	19.07
Right vein. μm	234	142.18	18.90
Average vein. μm	261	142.29	17.89
Major vein. μm	261	147.54	17.88
Left AVIx. μm	190	0.79	0.11
Right AVIx. μm	175	0.78	0.11
Average AVIx. μm	259	0.78	0.08

ABI = ankle brachial index; AVIx = arteriovenous index; BMI = body mass index; C- IMT = carotid intima media thickness; DBP = diastolic blood pressure; HbA1c = glycosylated hemoglobin; HDL = high-density lipoprotein; LDL = low-density lipoprotein; PP = pulse pressure; PWV = pulse wave velocity; SBP = systolic blood pressure; SD = standar deviation; VDP = voltage duration product. Artery, vein and AVIx values are retinal vessels.

**Table 3:** Characteristics of the VAV2 and VAV3 polymorphisms.

Gene	SNP ID	Base Change	SNP	Chr location	Assay ID	HWE
VAV2	rs602990	1780 T > C	p.Val594Met	9q34.1	C_2537403_10	>0.05
VAV3	rs7528153	c.892 T > A	Ser298Thr	1	C_447698_10	>0.05

Chr: chromosome; HWE: Hardy Weinberg equilibrium in control groups; SNP: single nucleotide polymorphism.

**Table 4:** Distribution of VAV3 rs7528153 genotypes among patients with or without pathological state.

SNP	Genotype	Pathological		Non pathological		P value	OR (95% CI)
		N	%	N	%		
VAV3 rs7528153	AA	144	53.9	47	38.5	Ref.	1.000
	AT	94	35.2	62	50.8	**0.002**	0.484 (0.304–0.770)
	TT	29	10.9	13	10.7	0.390	0.724 (0.346–1.514)
VAV3 rs7528153 dominant	AA	144	53.9	47	38.5	Ref.	1.000
	AT + TT	123	46.1	75	61.5	**0.004**	0.526 (0.338–0.817)
VAV3 rs7528153 recessive	AA + AT	238	89.1	109	89.3	Ref.	1.000
	TT	29	10.9	13	10.7	0.945	0.526 (0.338–0.817)

*P value & OR adjusted by sex and age. CI *=* confidence interval; OR *=* odd ratio; ref.* = *reference;* SNP = single nucleotide polymorphism.

**Table 5:** Distribution of VAV3 rs7528153 genotypes among patients with or without altered basal glycaemia.

SNP	Genotype	Altered basal glycaemia				P value	OR (95% CI)
		Yes		No			
		N	%	N	%		
VAV3 rs7528153	AA	24	49.0	167	49.1	Ref.	1.000
	AT	15	30.6	141	41.5	0.362	0.727 (0.367–1.442)
	TT	10	20.4	32	9.4	0.068	2.169 (0.944–4.985)
VAV3 rs7528153 dominant	AA	24	49.0	167	49.1	Ref.	1.000
	AT + TT	25	51.0	173	50.9	0.977	0.991 (0.543–1.807)
VAV3 rs7528153 recessive	AA + AT	39	79.6	308	90.6	Ref.	1.000
	TT	10	20.4	32	9.4	**0.024**	2.481 (1.129–5.453)

*P value & OR adjusted by sex and age. CI *=* confidence interval; OR *=* odd ratio; ref.* = *reference; SNP* = *single nucleotide polymorphism*.

**Table 6:** Distribution of VAV2 rs602990 genotypes among male patients with and without target organ damage.

SNP	Genotype	Target organ damage				P value	OR (95% CI)
		Yes		No			
		N	%	N	%		
VAV2 rs602990	CC	19	17.8	31	33.3	Ref.	1.000
	CT	63	58.9	40	43.0	**0.026**	2.253 (1.101–4.613)
	TT	25	23.4	22	23.7	0.298	1.559 (0.675–3.598)
VAV2 rs602990 dominant	CC	19	17.8	31	33.3	Ref.	1.000
	CT + TT	88	82.2	62	66.7	**0.044**	2.007 (1.018–3.956)
VAV2 rs602990 recessive	CC + CT	82	76.6	71	76.3	Ref.	1.000
	TT	25	23.4	22	23.7	0.746	0.894 (0.454–1.760)

*P value & OR adjusted by sex and age. CI = confidence interval*; *OR = odd ratio; ref.* = *reference;* SNP = single nucleotide polymorphism.

**Table 7:** Distribution of VAV2 rs602990 genotypes among obese (BMI > 30) and non-obese (BMI 25–30) patients.

SNP	Genotype	Obesity				P value	OR (95% CI)
		Yes		No			
		N	%	N	%		
							
VAV2 rs602990	CC	31	25.6	54	27.4	Ref.	1.000
	CT	52	43.0	102	51.8	0.814	0.935 (0.533–1.639)
	TT	38	31.4	41	20.8	0.109	1.679 (0.890–3.165)
VAV2 rs602990 dominant	CC	31	25.6	54	27.4	Ref.	1.000
	CT + TT	90	74.4	143	72.6	0.603	1.149 (0.682–1.936)
VAV2 rs602990 recessive	CC + CT	83	68.6	156	79.2	Ref.	1.000
	TT	38	31.4	41	20.8	**0.036**	1.753 (1.038–2.958)

*P value & OR adjusted by sex and age. CI *=* confidence interval; OR *=* odd ratio; ref.* = *reference; SNP* = *single nucleotide polymorphism*.

**Table 8:** Statistically significant results in the distribution of VAV2 rs602990 according to different cardiovascular characteristics measured on the patients.

Characteristic	VAV2 average ± SD			P value			VAV2 dominant average ± SD		P value	VAV2 recessive average ± SD		P value
	CC	CT	TT	CCvsTT	CCvsCT	CTvsTT	CC	CT + TT		CC + CT	TT	
DBP	83.01 ± 9.91	81.34 ± 10.73	79.28 ± 10.89	0.051	0.567	0.482	83.01 ± 9.91	80.61 ± 10.81	0.064	81.92 ± 0.61	79.28 ± 1.03	**0.045**
ABI right	1.13 ± 0.10	1.14 ± 0.09	1.11 ± 0.11	0.419	1.000	0.058	1.13 ± 0.10	1.13 ± 0.10	0.782	1.14 ± 0.10	1.11 ± 0.11	**0.043**
Retinal artery average	108.76 ± 11.07	111.19 ± 12.26	106.45 ± 13.43	0.988	0.454	**0.035**	108.76 ± 11.07	109.56 ± 12.39	0.609	110.33 ± 11.88	106.45 ± 13.43	**0.028**
Retinal artery minor	104.62 ± 11.14	106.74 ± 13.82	102.16 ± 14.18	1.000	0.747	0.076	104.62 ± 11.14	105.17 ± 14.08	0.733	105.99 ± 12.95	102.17 ± 14.18	**0.043**

*P value adjusted by age and sex. All data are corrected by Bonferroni post-hoc test. ABI *=* ankle-brachial index; DBP *=* *diastolic blood pressure; SD = standard deviation.
