# Corrigendum: Association of VAV2 and VAV3 polymorphisms with cardiovascular risk factors

**DOI:** 10.1038/srep46977

**Published:** 2018-05-11

**Authors:** Nuria Perretta-Tejedor, Javier Fernández-Mateos, Luis García-Ortiz, Manuel A. Gómez-Marcos, José I. Recio-Rodríguez, Cristina Agudo-Conde, Emiliano Rodriguez-Sánchez, Ana I. Morales, Francisco J. López-Hernández, José M. López-Novoa, Rogelio González-Sarmiento, Carlos Martínez-Salgado

Scientific Reports 7: Article number: 4187510.1038/srep41875; published online: 02 03 2017; updated: 05 11 2018

This Article contains typographical errors in the Acknowledgements section.

“This work was supported by grants from Instituto de Salud Carlos III (Ministry of Economy and Competitiveness, PI12/00959, PI13/01741, RETICS RD012/0005/004 and RD012/0021, co-funded by FEDER) and Junta de Castilla y León (Excellence Group GR100, Ministry of Health, BIO/SA87/13 and IES095U14) and Fundación Mutua Madrileña (IX Call for Grants and Aids for Medical Research).”

should read:

“This work was supported by grants from Instituto de Salud Carlos III (Ministry of Economy and Competitiveness, PI15/01055, PI13/01741, RETICS RD012/0005/004 and RD012/0021, co-funded by FEDER) and Junta de Castilla y León (Excellence Group GR100, Ministry of Health, BIO/SA87/13 and IES095U14) and Fundación Mutua Madrileña (IX Call for Grants and Aids for Medical Research).”

